# Health equity in Lebanon: a microeconomic analysis

**DOI:** 10.1186/1475-9276-9-11

**Published:** 2010-04-14

**Authors:** Nisreen Salti, Jad Chaaban, Firas Raad

**Affiliations:** 1 Department of Economics, American University of Beirut, PO Box 110236, Riad el Solh, Beirut, 11072020, Lebanon; 2 Department of Agricultural Sciences, American University of Beirut, PO Box 110236, Riad el Solh, Beirut, 11072020, Lebanon; 3 Senior Health Specialist and Human Development Coordinator/GCC Countries Middle East and North Africa Region (MENA) The World Bank - 1818 H St. N.W. Washington, D.C. 20433, USA

## Abstract

**Background:**

The health sector in Lebanon suffers from high levels of spending and is acknowledged to be a source of fiscal waste. Lebanon initiated a series of health sector reforms which aim at containing the fiscal waste caused by high and inefficient public health expenditures. Yet these reforms do not address the issues of health equity in use and coverage of healthcare services, which appear to be acute. This paper takes a closer look at the micro-level inequities in the use of healthcare, in access, in ability to pay, and in some health outcomes.

**Methods:**

We use data from the 2004/2005 Multi Purpose Survey of Households in Lebanon to conduct health equity analysis, including equity in need, access and outcomes. We briefly describe the data and explain some of its limitations. We examine, in turn, and using standardization techniques, the equity in health care utilization, the impact of catastrophic health payments on household wellbeing, the effect of health payment on household impoverishment, the equity implications of existing health financing methods, and health characteristics by geographical region.

**Results:**

We find that the incidence of disability decreases steadily across expenditure quintiles, whereas the incidence of chronic disease shows the opposite pattern, which may be an indication of better diagnostics for higher quintiles. The presence of any health-related expenditure is regressive while the magnitude of out-of-pocket expenditures on health is progressive. Spending on health is found to be "normal" and income-elastic. Catastrophic health payments are likelier among disadvantaged groups (in terms of income, geography and gender). However, the cash amounts of catastrophic payments are progressive. Poverty is associated with lower insurance coverage for both private and public insurance. While the insured seem to spend an average of almost LL93,000 ($62) on health a year in excess of the uninsured, they devote a smaller *proportion *of their expenditures to health.

**Conclusions:**

The lowest quintiles of expenditures per adult have less of an ability to pay out-of-pocket for healthcare, and yet incur healthcare expenditures more often than the wealthy. They have lower rates of insurance coverage, causing them to spend a larger proportion of their expenditures on health, and further confirming our results on the vulnerability of the bottom quintiles.

## Background

Out-of-pocket spending on health is a major concern for policymakers, especially in developing countries where direct household payments for health care can account for the single largest component of household spending after food expenditures. High private health spending is related to the incidence of illnesses and chronic diseases, but it can grow exponentially and affect the living conditions of individuals in situations of crisis, conflict and natural disasters. These catastrophic health payments can push households into poverty or into deeper poverty. Households facing these health expenses may cut back on other essential household spending such as food and clothing. Households may also reduce their consumption of healthcare services, thus causing the health condition of family members to further deteriorate. To date, there has been little work done on the level and distribution of household out-of-pocket payments for health care in the Middle East, and to what extent household expenditures on health care affect living standards. Given that the region is prone to recurrent conflict and political instability, it becomes very important to determine who the most vulnerable groups with regards to healthcare access and financing are. We focus in our analysis on the Lebanese economy, where health equity issues have not yet been thoroughly studied.

**Figure 1:**
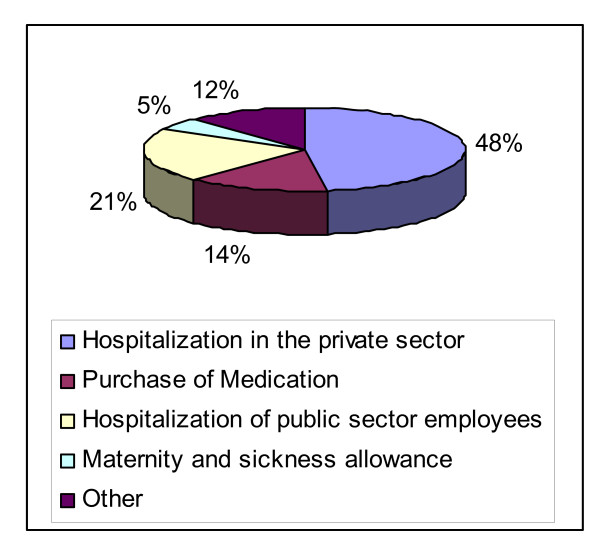
**Distribution of public health expenditure (2006)**. Source: Ministry of Finance 2006 budget proposal.

The Lebanese healthcare system suffers from high spending: almost 8.8% of GDP compared to an average of 5% in the MENA region, of which 54% is private expenditure, 75% of those out-of-pocket. Most spending is geared towards hospital-based curative care, as the public sector expenditures on hospitalization accounts for more than 75% of total public health spending in 2006 [1]. Private sector hospitalization accounts for 48% of total public health expenditure, which constitutes a significant drain on public sector finances (Figure 1). Despite high spending, hospitals in Lebanon are still characterized by a low occupancy rate in public hospitals (56 percent in 2004 compared to an OECD average of 80 percent). The allocation of total hospital beds, which stands at 36 beds per 10,000 people, is highly skewed towards the capital and its suburbs, as these areas show a ratio of almost 70 beds per 10,000; while the poorer regions of South and Eastern Lebanon have a ratio below 20 beds per 10,000 [2]. In addition, health services in Lebanon are some of the most expensive in the region.

One of the structural weaknesses in the Lebanese health care system is related to the fact that the role of the Ministry of Health has focused almost exclusively on the provision of services, while its role in prevention, planning and regulation remained limited. This is particularly true in light of the expanding role of the private sector (Figure 1). Both these factors also explain the prevalence of the more costly and arguably less effective culture of curative care rather than the more efficient strategy of preventive care in the Lebanese healthcare system.

Recognizing the inefficiency of the health care sector in Lebanon, the World Bank initiated with the Lebanese Government under the Paris III reform framework a Social Protection Development Policy Loan (DPL) including several health sector reforms. The loan package comprises key reforms in the health insurance sector, measures to rationalize health expenditures, and critical reforms in public health policy. The public health reforms supported by the DPL consist of:

i. Implementation, by the Ministry of Health of a health expenditure rationalization plan to better contain rising hospitalization expenditures.

ii. Reform initiative to revitalize primary healthcare through the implementation of a five-year action plan based on a recently developed primary healthcare strategy for the country.

iii. Reform initiative to fully develop and operationalize the national Expanded Program on Immunization (EPI).

iv. Reform initiative to significantly upgrade the core public health function of disease surveillance.

All these reforms are intended to contain the fiscal waste caused by high and inefficient public health expenditures (Table 1), in order to render the Lebanese healthcare system more cost-effective. Yet these reforms do not directly address the issues of health equity and coverage of healthcare services, which appear quite acute in Lebanon. These reforms are untargeted and do not specifically focus on communities with low access to healthcare and health insurance. In this paper, we propose a different reform approach than the one suggested by the World Bank, by focusing on health inequity.

**Table 1:** Public sector health spending (2006)

*Health expenditure item*	2006 Budget Proposal (000 LL)	as a % of total budgetary expenditures	as a % of GDP
Hospitalization in the private sector	240,725,000	2.15%	0.71%
Purchase of Medication	73,156,500	0.65%	0.21%
Hospitalization of public sector employees	105,500,000	0.95%	0.31%
Maternity and sickness allowance	25,600,000	0.23%	0.08%
Other	60,064,332	0.53%	0.18%

**Total**	**505,045,832**	**4.50%**	**1.48%**

Source: Ministry of Finance 2006 Budget Proposal

Total public health spending in 2006, which includes the budgets of the Civil Servants' Cooperatives, the National Social Security Fund (NSSF) and the allowances of the Ministries of the Displaced and Education allowances.

Health inequity can be defined as "a particular type of difference in health or in the most important influences on health that could potentially be shaped by policies; it is a difference in which disadvantaged social groups (such as the poor, racial/ethnic minorities, women, or other groups that have persistently experienced social disadvantage or discrimination) systematically experience worse health or greater health risks than more advantaged groups" [3]. Thus, inequity in health reflects the systematic differences across socio-economic groups in one or more aspects of health [4]. While the definition we use more closely matches the definition of health equity as equality in health, it is also consistent with the understanding of health inequality as an indicator of general injustice in society [5,6].

In recent years, rising global interest in the area of health equity has spawned numerous international research and policy initiatives [7]. These initiatives have largely focused on measuring and explaining inequalities in health status outcomes (infant mortality or maternal mortality), health service use (antenatal care visit), and public subsidies supporting health service utilization. Policy initiatives have sought to alleviate disparities by addressing modifiable factors through organizational, economic and/or regulatory reforms. Another focal area of health equity research has revolved around the progressivity of health care payments and the catastrophic and impoverishing impact of these payments on individual households [7]. A number of global evaluations mainly conducted by the WHO have highlighted the variations in household payments for health care and their implications, shedding light on the international focus afforded to the issue of financial protection in health care [8].

This paper takes a closer look at the micro-level inequities in the use of healthcare, in access to healthcare, in the ability to pay for it, and in some health outcomes. The findings contribute to the international literature on health inequity by providing more evidence from Middle Eastern countries. The results of our analysis are especially useful for policymakers in Lebanon and other countries that face the issue of promoting access to health services by targeting the most needy, while at the same time maintaining efficiency and financial stability in volatile security environments.

In the methods section of this paper we briefly describe the data and explain our choice of measures. We also explain in several subsections the methods used, including two techniques for standardization of our use measures. We describe, in turn, our measures of health care utilization, of catastrophic health payments, of existing health financing methods. In the results and discussion section, we illustrate our major findings regarding the equity of healthcare use, the incidence of catastrophic health payments, the impoverishing effect of healthcare spending, the distribution of health financing methods and a geographical breakdown of health characteristics in the country. We also qualify our results with a discussion of the limitations of our data and our approach. In the conclusion section, we summarize our results and present policy recommendations based on our findings.

## Methods

### Data description

This paper uses micro-data from the 2004/2005 Multi-Purpose Survey of Households conducted by the United Nations Development Programme (UNDP), the Ministry of Social Affairs (MoSA) and the Central Administration for Statistics (CAS). This is the most recent national survey of household living conditions to be conducted in Lebanon. The survey collects data on socio-demographics, household characteristics (including data on expenditures, assets and geographical characteristics), labor market characteristics and some health variables. The data contain information on close to 56,000 individuals from 13,000 households in all 6 Lebanese mohafazas (governorates). The survey focuses exclusively on Lebanese nationals and therefore excludes other residents in Lebanon (Palestinian refugees, foreign migrant workers, etc.).

Access to raw data from the survey was secured through MoSA, however all income data, including variables measuring financial assistance in healthcare from the government or non-profit sectors, remain inaccessible, as the Central Administration for Statistics has yet to release the income measures from the survey. The lack of access to income data constrains our ability to conduct welfare analysis. As has become standard practice in the literature, we use the distribution of household expenditures per adult equivalent in our investigation of equity in access and use. Adult equivalent scaling follows the OECD scale of weighing as 1 count the first adult and as 0.7 every subsequent adult, and as 0.5 count every individual under age 15 in the household. Our poverty line is also expenditure based, as are some of our variables measuring the use of healthcare services. This presents some technical difficulties in the analysis of the poverty implications and impoverishing effects of healthcare expenditures.

### Measurement of variables

#### a. Need variables

We define three need variables based on the availability of health outcome measures in the household survey. These include indicator variables for:

1. the presence of a disability

2. the presence of a chronic condition

3. being a mother of a newborn child (within the last 12 months) as we take childbearing to be an indicator of the need for some healthcare

A list of the disabilities and chronic conditions included is provided in additional file 1. We include all three of these variables in our standardizations (described below).

#### b. Use variables

We define two broad classes of household-level use variables (which are then scaled to household size to get per-capita measures):

1. An indicator variable measuring the presence of any health related expense, including spending on health insurance.

2. A variable measuring the dollar amounts of health care spending.

The reason we are able to use the presence of any health related expense as an indicator of use is that no health care financing plans in Lebanon involves complete coverage of health expenses, with the exception of the government provided insurance plan for military and security personnel. For all other Lebanese citizens, even the most generous coverage involves some out-of-pocket expenditures, so we take the presence of health-related spending as an indicator of the use of healthcare services [9].

#### c. Catastrophic health payments

We then look at the incidence of catastrophic health payments (payments over 25 percent of expenditures per adult equivalent) across different population groups and socioeconomic characteristics.

#### d. Insurance coverage

We identify two broad classes of insurance plans: publicly provided plans (the National Social Security Fund, or NSSF, the Civil Servants' Cooperatives, municipal government plans, and plans of the security and armed forces) and privately provided plans (for the employed, the self-employed and the syndicated).

### Methods used

#### a. Means and concentration indexes

The methods that are employed to quantify the degree of equity in health care include descriptive and regression techniques using national household survey data described above. Descriptive methods include comparison of means and the 'concentration index' technique. The concentration index is a measure of how equally a health variable is distributed across a population ranked by income level. As a single numeric, the concentration index allows degrees of equity to be easily captured and compared, in order to determine the extent and nature of policy reform that is necessary.

#### b. Standardization: direct, indirect

A proper assessment of the equity of healthcare must control through regression analysis for the confounding effects of need and demographics, as well as other sources of heterogeneity that might affect healthcare use. We standardize our measures of the use of healthcare on three measures of need, as well as the main demographics we take to be correlated to utilization, using both the direct and indirect standardization techniques [8].

Direct standardization predicts the distribution of use by expenditure quintile that would be observed if the distribution of the confounding variables (need, age and sex) was the same for each quintile, but confounding variables had quintile-specific effects. Indirect standardization involves predicting the value of use in the same way, using the observed values for the confounding variables, but constraining their effect on use to be the same across all quintiles. In standardizing, we control for health confounding effects such as age, gender, and non-confounding effects such as education, the log of household expenditures and employment.

#### c. Methodology in poverty analysis

Our analysis of catastrophic health payments involves the calculation of headcounts and overshoots. The un-weighted headcount treats all households equally in calculating the share of households that incur a catastrophic health payment. This statistic assumes constant marginal utility of income. A measure that is more sensitive to equity concerns and that assigns more weight to households at the bottom of the distribution is the rank-weighted headcount. The concentration index measures the degree of equity in the incidence of catastrophic health payments across the income distribution and a negative index is indication that households at the lower end of the distribution of total household expenditures have a greater tendency to exceed the spending threshold on health and incur catastrophic health payments [7].

The depth of the impact of catastrophic payments is calculated using the overshoot, which calculates the average excess above the threshold. Like the headcount, the un-weighted version of the overshoot treats any dollar in excess of the threshold equally, regardless of which household is spending it. The rank-weighted overshoot instead assigns greater weight to overshoot spending by households at the bottom of the expenditure distribution. The concentration index measures the equity of the overshoot across the distribution of household expenditures: a positive value indicates that the overshoot tends to be greater among the better off.

When it comes to the analysis of poverty, we are unable to capture the impoverishing effects of healthcare spending (particularly when its scale is catastrophic) when our poverty line is defined on the basis of expenditure levels: the larger an individual's spending on healthcare, the likelier the individual's overall expenditures exceed the poverty line, which leads to an artificially lower poverty headcount. One approach that is commonly used to address this difficulty in constructing a valid measure of poverty is to calculate net poverty rates and net poverty gaps, which include only non-health spending [7]. The weakness of this approach is that insofar as the poverty line is constructed to include spending on healthcare, net figures will overstate poverty. Another approach that we propose is to extrapolate from the overall poverty line and the composition of the mean household's spending a poverty line for non-health expenditures. Poverty rates and gaps calculated based on this line are not affected by the extent of healthcare spending, nor should the threshold itself include expenditures on health. This method is similar in essence to the one developed by Wagstaff et al. (chapter 19) [7]. One of the major limitations in this approach is the arbitrariness of choosing the breakdown in the expenditures of the mean household to extract from the poverty line the amount of spending on non-health related goods.

### Data limitations

Because health expenditures are not disaggregated into different classes or types of healthcare goods and services, we have no information on the nature of healthcare consumption. Thus, for example we have no means of discerning publicly provided healthcare from health services that are privately provided. Similarly, we cannot distinguish between inpatient, outpatient and specialist care.

Another limitation of the household survey is the paucity of variables measuring health outcomes. Disabilities and chronic conditions are recorded; however data on health status, whether self-assessed or measured by a healthcare professional, were not collected in this survey. Furthermore, unlike many surveys of the living conditions of households, this survey fails to record recent illness or injury, recent visits to healthcare centers or the recent use of the services of a healthcare professional.

## Results and Discussion

### Distribution of need

Table 2 presents a breakdown of the first two of our need variables (the presence of a disability and the presence of a chronic condition) across quintiles of expenditures per adult equivalent (Table 2).

**Table 2:** Need across expenditure quintiles

	Poorest	2	3	4	Richest	Total
%of quintile with disability	2.9%	2.0%	2.2%	1.7%	1.2%	2.0%
%of quintile with disability ind. std.	3.0%	2.0%	2.2%	1.7%	1.2%	2.0%
%of quintile with disability dir. std.	3.3%	2.1%	2.3%	1.6%	1.2%	2.1%

% of quintile with chron. cond.	14.3%	16.0%	16.4%	18.3%	18.6%	16.7%
% of quintile with chron. cond. ind. std.	15.9%	16.8%	16.8%	16.8%	16.4%	16.5%
% of quintile with chron. cond. dir. std.	16.5%	17.7%	17.6%	17.6%	17.3%	17.3%

Source: Authors' estimate from 2004/2005 Household Survey

The incidence of disability decreases steadily across expenditure quintiles moving from close to 3% for the poorest fifth to 1.2% for the richest quintile. When standardized on age and gender whether directly or indirectly, these figures become slightly higher than the non-standardized figures particularly for the poorer quintiles. The incidence of chronic disease across quintiles shows the opposite trend to the one for disabilities: the incidence of chronic disease increases monotonically from 14.3% in the poorest quintile to 18.6% in the top quintile. This difference may be due to a difference in the frequency and accuracy of diagnoses that the various expenditure classes have access to. When standardized on age and gender, the differences across quintiles shrink somewhat as the standardized figures are slightly higher for poorer quintiles and lower for richer ones.

### The distribution of health care expenditures

#### a. The incidence of health-related expenses

We report measures of the incidence of health-related expenses, including spending on health insurance (Table 3). The three columns of the table report the average incidence by expenditure quintile, for the un-standardized incidence of health-related expenses, its indirectly standardized measure and its directly standardized measure. Even controlling for our three measures of need as well as other correlates of healthcare spending, Table 3 show that the incidence of health related expenditures is regressive: holding fixed the presence of a disability or a chronic condition, the recent birth of a child, age, gender, education and employment, and estimating the predicted incidence of the presence of any healthcare spending by expenditure quintile shows that the poorest quintile is less likely to spend on health care, which we take here to be an indication of a lower level of healthcare use. However, the probability of healthcare spending doesn't increase monotonically across expenditure quintiles: the second quintile is more likely to "consume" healthcare than are the next two, and the third and fourth quintiles have almost identical health consumption probabilities. The single largest gap separates the poorest quintile from the next to poorest, for all three measures. In the absence of any direct measure of income, a measure of household expenditure is used as a control variable. The results are qualitatively unchanged when only non-health expenditures are used as a control.

**Table 3:** Incidence of health related expenditures by expenditure quintile

*Quintile*	Observed	**Indirectly Stand**.	**Directly Stand**.
Poorest	0.85	0.46	0.85
2	0.91	0.53	0.91
3	0.90	0.52	0.90
4	0.90	0.52	0.90
Richest	0.93	0.55	0.93

Total	0.90	0.51	0.90

Source: Authors' estimate from 2004/2005 Household Survey

We note the remarkable difference between the indirectly standardized measures and the observed directly standardized rates of healthcare use. Thus, when overall means of the non-confounding variables are used (in indirect standardization), predicted healthcare spending is much lower for all five quintiles than if we assumed quintile means for household expenditures, employment and education. The absolute difference in predicted usage rates across quintiles is roughly the same for all three measures, which makes the difference proportionately much larger when use is indirectly standardized. This is the result of the substantial differences in the non-confounding variables (expenditures, employment and education) across quintiles.

#### b. Out-of-pocket expenditures on health

We also look at average out-of-pocket expenditures (excluding insurance payments) on health by expenditure quintile (Table 4). The pattern is very similar to that of the incidence of health related payments: out-of-pocket expenditures increase with expenditure quintiles, even when standardized on need, age and sex and controlling for total household expenditures, employment and education. The largest proportional increase is between the average out-of-pocket expenditures on health of the 4^th ^quintile and that of the top fifth of the expenditure distribution, as both observed and standardized expenditures of the highest spending quintile are over double those of the 4^th ^quintile.

**Table 4:** Out-of-pocket health related expenditures by expenditure quintile

*Quintile*	Observed (000 LL)	**Indirectly Stand**. (000 LL)	**Directly Stand**. (000 LL)
Poorest	73	85	75
2	149	159	153
3	216	224	219
4	361	357	353
Richest	756	750	730

Total	309	313	303

Source: Authors' estimate from 2004/2005 Household Survey

**Figure 2:**
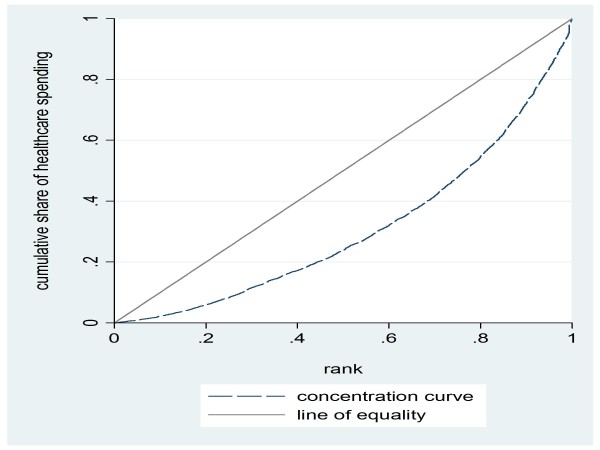
**Concentration curve for healthcare spending**. Source: Authors' estimates using 2004/2005 Household Survey

To further examine the equity of average out-of-pocket expenditures on health, we construct concentration curves (Figure 2), which show the cumulative share of healthcare spending against the distribution of expenditures per adult equivalent. The curve shows a substantial gap from the equality line which is also reflected in the corresponding concentration index of 0.676. Out-of-pocket health expenditures appear to be progressive.

**Figure 3:**
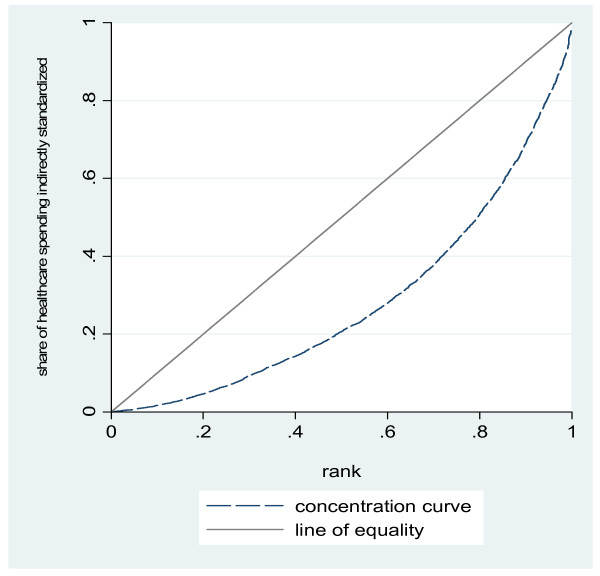
**Concentration curve for healthcare spending, indirectly standardized**. Source: Authors' estimates using 2004/2005 Household Survey

We reconstruct the concentration curve for out-of-pocket expenditures once they have been indirectly standardized on need (Figure 3). Indirectly standardized expenditures appear to be substantially more regressive than observed health expenditures: once need and relevant demographics are accounted for, the resulting measure of out-of-pocket expenditures on healthcare shows serious inequity in the healthcare spending across the income distribution.

#### c. The disability card

A service with much narrower subscription and more limited use is the disability card provided by MoSA. The program is called "Rights and Access Program for the Disabled". Its objective is to ensure that persons with disabilities are self-reliant, can live independent lives and are integrated into society. During the last 10 years, the program has issued disability cards to 60,000 persons, 22,000 of whom have been provided technical aid [10]. Each person with disabilities registered with the program is assessed for needs and the system has a database of needs for services. Card holders are eligible for specialized aid and services at accredited institutions, yet for the past couple of years the program has faced many difficulties due to shortage in financing [10]. Only 1,150 survey respondents answer the disability card question, which is the number of respondents who report having a disability. Of these, 423 respondents hold a disability card, and only 176 have used it in the past year. The distribution of the use of the services provided by the disability card (Table 5) shows that the observed incidence of use does not increase monotonically. However, once this use variable is standardized, it increases with expenditure per adult equivalent. Higher household expenditures are associated with a higher likelihood of using the disability card.

**Table 5:** Use of the MOSA disability card

*Quintile*	Observed	**Indirectly Stand**.	**Directly Stand**.
Poorest	3‰ 6,624	1‰ 6,624	2‰ 6,155
2	2‰ 6,744	2‰ 6,744	2‰ 6,236
3	4‰ 6,432	3‰ 6,432	3‰ 5,976
4	4‰ 6,558	4‰ 6,558	4‰ 6,078
Richest	3‰ 6,059	4‰ 6,059	5‰ 5,536

Total	3‰ 32,417	3‰ 32,417	3‰ 29,981

Source: Authors' estimate from 2004/2005 Household Survey

The use of healthcare services, whether measured by the presence of any health-related expense, the use of a disability card to access government provided health services, or the value of out-of-pocket expenditures on health appears to be regressive when measured against the distribution of overall household expenditures per adult equivalent. This result holds for the observed values of healthcare use as well as values standardized on need and other health determinants. Our results for equity in need showed disparity in the patterns of each need variable, but the more prevalent of our two need variables (the presence of a chronic health condition) put upper expenditure quintiles at more of a disadvantage. The results we find for use, even when standardized for need show patterns that consistently favor the rich.

Among the disabled for whom we have expenditures data, we report the quintile distribution of those holding a disability card (Table 6).

**Table 6:** Ministry of Social Affairs disability card by expenditure quintile

*Quintile*	Observed	**Indirectly Stand**.	**Directly Stand**.
Poorest	3‰ 6,624	1‰ 6,624	2‰ 6,155
2	2‰ 6,744	2‰ 6,744	2‰ 6,236
3	4‰ 6,432	3‰ 6,432	3‰ 5,976
4	4‰ 6,558	4‰ 6,558	4‰ 6,078
Richest	3‰ 6,059	4‰ 6,059	5‰ 5,536

Total	3‰ 32,417	3‰ 32,417	3‰ 29,981

Source: Authors' estimate from 2004/2005 Household Survey

### The impact of catastrophic health payments

As a background to the discussion of catastrophic health payments and their impact on welfare, we look at the breakdown across expenditure quintiles of the share of healthcare in household expenditures as well as the share of healthcare in non-food expenditures. We also look at the breakdown of the characteristics of individuals making catastrophic payments [11]. The characteristics that we look at are gender, geographical regions (mohafaza) and age.

We analyze the effect of health payments on household impoverishment, including accounting, under various assumptions, for the number of households who fall under the poverty line due to health payments. We find that health's share in expenditures increases monotonically across quintiles, and that the proportion of non-food expenditures spent on health increases most when we move from the first to the second quintile and from the third to the fourth quintile (Table 7). This preliminary look is an indication that spending on health seems to be "normal" (where expenditure is a proxy for income) and expenditure-elastic as spending on healthcare must increase more than proportionally with overall expenditures for the share of health to be rising over quintiles.

**Table 7:** Share of health in expenditures by quintile

*Quintile*	Health share in expenditures	Health share in non-food expenditures
Poorest	4.8%	6.9%
2	6.3%	8.7%
3	6.5%	8.5%
4	7.5%	9.4%
Richest	8.2%	9.7%

Total	6.6%	8.6%

Source: Authors' estimate from 2004/2005 Household Survey

#### a. Who incurs catastrophic health payments?

**Figure 4:**
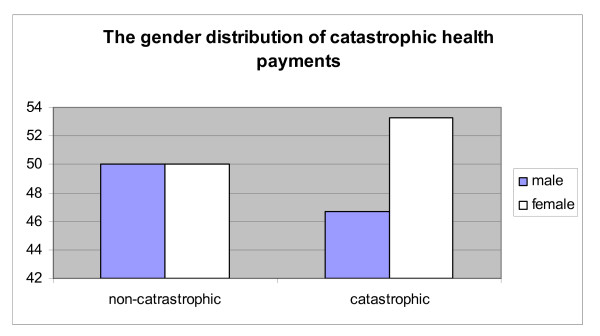
**The gender distribution of catastrophic health payments**. Source: Authors' estimates using 2004/2005 Household Survey

Unsurprisingly, the gender breakdown of individuals making catastrophic health payments (Figure 4) is strongly skewed towards women (where 53% of people incurring catastrophic health payments are women, whereas women are only 50% of individuals not making catastrophic health payments). The difference in the incidence of catastrophic payments by gender is significant at the 1% level.

**Figure 5:**
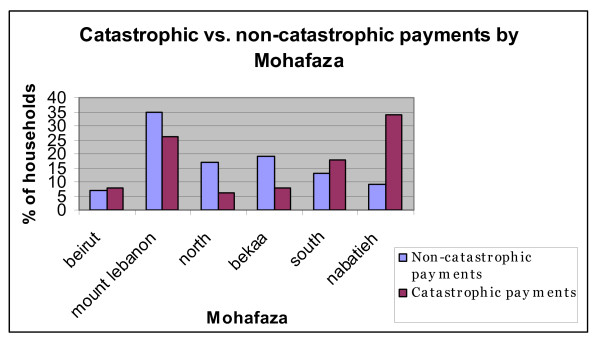
**Catastrophic vs. non-catastrophic payments by Mohafaza (province)**. Source: Authors' estimates using 2004/2005 Household Survey

We compare the geographical breakdown of households making catastrophic healthcare payments to that of households whose expenditure on healthcare does not exceed 25% of their overall household expenditures (Figure 5): the sharpest difference in the distribution is in the mohafaza of Nabatieh which accounts for around 9% of households whose healthcare payments fall below 25% of total household expenditures but close to 35% of households incurring catastrophic healthcare. The mohafaza of the South also shows sharp contrast: around 13% of households with non-catastrophic payments and close to 18% of households straddled with healthcare payments. Other remarkable differences that deserve note are that about 35% of households who spend less than a quarter of their income on healthcare are in Mount Lebanon. Similarly, the otherwise quite poor mohafaza of the North houses 17% of households whose payments are non-catastrophic but just 6% of households making catastrophic health payments. These results show an overlap between the districts that exhibit the starkest contrast in catastrophic healthcare payments and the incidence of repeated episodes of political violence (mainly in the governorates of Nabatieh and South Lebanon, in the southern part of the country) and their legacy of liabilities in terms of lifetime disabilities and risks in terms of unexploded mines and munitions.

**Figure 6:**
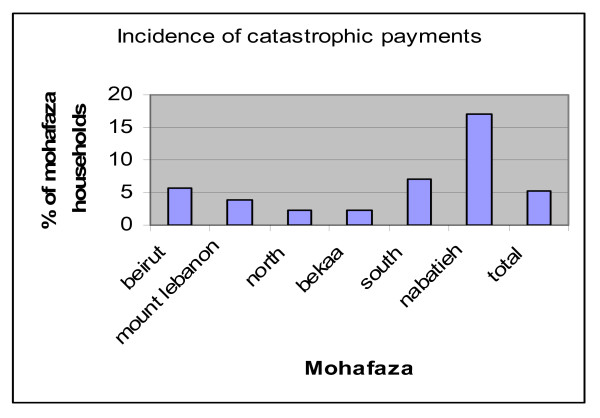
**Incidence of catastrophic payments by Mohafaza (province)**. Source: Authors' estimates using 2004/2005 Household Survey

We also look at the incidence of catastrophic payments per mohafaza (Figure 6) and find that Nabatieh has, by far, the highest risk of catastrophic health payments (17%), the South is a distant second (7%).

Individuals who face catastrophic health payments are unsurprisingly significantly older (15 years) than those who don't. Households faced with catastrophic health payments tend to be slightly smaller by close to one person on average (Table 8).

**Table 8:** Characteristics of individuals facing catastrophic-health payments

	Non-catastrophic (health payments < 25% of expenditures)	Catastrophic (health payments >= 25% of expenditures)
Age	29.4	40.5
Household Size	4.5	3.5

Source: Authors' estimate from 2004/2005 Household Survey

We put all of these effects together in a logistic regression of the probability of incurring catastrophic health payments so we can control for all the confounding effects including gender, age, mohafaza, need, insurance coverage and insurance type (Table 9). We find that log expenditures per adult equivalent are associated with significantly higher log odds of catastrophic payments, as is age. Two of our need variables (the presence of a disability and the presence of a chronic condition) are associated with significantly higher odds of catastrophic payments. All public insurance plans are associated with lower probability of catastrophic health payments, as is private health insurance that is self-paid or syndicate provided. The omitted governorate is Beirut, the capital, and we find that the Bekaa and the North have, on average, a significantly lower incidence of catastrophic health payments (at 1 and 10% significance, respectively), whereas the governorates of the South and Nabatieh have significantly higher risk of catastrophic health payment, as the results of the bivariate analysis in Figure 6 also indicates.

**Table 9:** The determinants of catastrophic health payments.

Dependent variable: Presence of catastrophic health payments N = 13,944	Logistic regression
Log household expenditures per adult equivalent	0.50** (0.08)
Age 0-15	-1.73** (0.17)
Age 16-25	-1.35** (0.18)
Age 26-45	-1.51** (0.15)
Age 46-65	-1.15** (0.14)
Disability	0.70** (0.25)
Chronic condition	0.79** (0.12)
Presence of a recent mother	-0.36 (0.36)
Male	-0.12 (0.09)
NSSF	-1.98** (0.21)
Coop	-2.23** (0.25)
Insurance (Army)	-2.20** (0.23)
Private insurance (employer)	0.23 (0.27)
Private insurance (own)	-1.58** (0.37)
Private insurance (mutual)	0.98** (0.32)
Private insurance (syndicate)	-1.62** (0.40)
Public insurance	1.67** (0.26)
Mount Lebanon	-0.01 (0.16)
North	-0.40^+^ (0.23)
Bekaa	-1.16** (0.25)
South	0.77** (0.18)
Nabatieh	1.52** (0.26)

Source: Authors' estimate from 2004/2005 Household Survey

#### b. Welfare effects of catastrophic health payments

Table 10 shows the un-weighted headcount, its concentration index and a rank-weighted headcount, a measure of the overshoot, its concentration index and a rank-weighted overshoot for households that incur catastrophic health payments. Rank-weighted measures introduce a normative interpretation to the headcount and overshoot as it attaches more importance to the contribution of poorer households to these indexes. The rank-weighted headcount is also the headcount multiplied by the complement of the concentration index [12]. These metrics are computed under 5 different values of the threshold for catastrophic payments (5%-25% in increments of 5%) and under 3 different values of the threshold for health expenditures out of non-food expenses. The last line of the table reports the mean positive overshoot which measures the intensity of the overshoot for households that face catastrophic health payments.

**Table 10:** Percentage of households incurring catastrophic payments for healthcare

	Share of overall expenditures				Share of non-food expenditures			
	5%	10%	15%	20%	25%	15%	25%	40%

Head Count	23.3%	13.3%	8.4%	5.6%	3.7%	11.8%	6.0%	2.3%

Concentration Index	-0.28	-0.26	-0.24	-0.22	-0.21	-0.28	-0.26	-0.21

Weighted Head Count	30.0%	16.8%	10.5%	6.8%	4.5%	15.2%	7.5%	2.8%

Overshoot	4.3%	2.8%	1.8%	1.2%	0.8%	3.0%	1.5%	0.5%

Concentration Index	0.13	0.16	0.19	0.23	0.27	0.10	0.15	0.25

Weighted Overshoot	3.8%	2.3%	1.5%	0.9%	0.6%	2.7%	1.3%	0.4%

Mean Positive Overshoot	18.5%	20.8%	21.8%	22.3%	22.4%			

Source: Authors' estimate from 2004/2005 Household Survey

Table 10 shows that the concentration index of headcounts is consistently negative, indicating that households at the bottom of the distribution are more likely to incur catastrophic health payments, using a variety of different possible thresholds for catastrophic payments. This might be the result of a rigid payment structure that is not a function of income. The concentration index is greatest for the lowest value of the threshold, which thereby results in the biggest discrepancy between the weighted and un-weighted measures of the headcount.

Interestingly, however, the concentration index of overshoots is positive indicating that while poorer households are more at risk of catastrophic payments, the dollar amounts of these payments is progressive: the better off tend to face larger overshoots. This result may be driven by non-linearity in health consumption which requires a minimal level of health spending regardless of total expenditure, but then increases more than proportionately as total expenditures increase.

Using only non-food expenditures (and adjusting the thresholds accordingly) gives qualitatively similar results in terms of the analysis of progressivity and poverty impact: head counts have a negative concentration index which makes the discrepancy between un-weighted and weighted headcounts largest when the threshold is lowest. Overshoots are progressive.

### The impoverishing effect of healthcare spending

#### a. Poverty analysis using national and World Bank poverty lines

Conventional poverty analysis is conducted using 4 different poverty lines: a deep national poverty line equivalent to expenditures of $2.2 per person per day, a national poverty line of $4 per person per day, the deep poverty line of $1 (or 1.08 in PPP) and the poverty line of $2 (or 2.15 in PPP) used by the World Bank, keeping in mind that the rate of exchange of the dollar to the Lebanese Pound is LL1,500/$. Poverty rates are calculated as a headcount of individuals lying below the poverty line, as a fraction of the overall population. Poverty gaps are calculated as the average shortfall from the poverty line (per year). And normalized poverty gaps express poverty gaps as a percentage of the poverty line. For poverty rates, poverty gaps and normalized poverty gaps, we calculate both gross and net measures, where health expenditures are included in the gross measures and netted out in the net measures.

In Table 11 we report the figures from the poverty analysis described above. Excluding healthcare from household resources increases the poverty headcount by only about a percentage point for the deep poverty line and by about 4 percentage points for the poverty line for national figures for poverty lines and by less than that for World Bank lines. More substantial differences in poverty rates come from comparing national to World Bank figures: the poverty line for Lebanon gives a gross poverty rate of around 27% whereas the same rate is only around 5% when the World Bank poverty line is used. The gross poverty gap is on the order of LL167,000 a year if we use the national poverty line, the net poverty gap is closer to LL200,000, and the poverty gap is around 8% of the poverty line. The calculated gaps are much narrower using World Bank poverty lines which show much lower poverty rates.

**Table 11:** Measures of poverty based on consumption gross and net of spending on health care

	Gross of health payments (1)	Net of health payments (2)	Difference	
			
			Absolute (3) = (2)-(1)	Relative [(3)/(1)] × 100
**$2.2 per day poverty line**				
Poverty headcount	5.3%	6.4%	1.1%	20.7%
Poverty gap (LL)	11,389	14,508	3,119	27.4%
Normalized gap	1%	1.2%	0.2%	20%
**$4.4 per day poverty line**				
Poverty headcount	27.5%	31.6%	4.1%	14.9%
Poverty gap (LL)	167,061	197,710	30,649	18.3%
Normalized gap	7.6%	9.0%	1.4%	18.4%
**$1.08 per day poverty line (WB)**				
Poverty headcount	0.2%	0.2%	0%	0%
Poverty gap	184	235	51	27.7%
Normalized gap	0.03%	0.04%	0.01%	33.3%
**$2.15 per day poverty line (WB)**				
Poverty headcount	5.0%	6.0%	1%	20%
Poverty gap	10,000	12,821	2,821	28.2%
Normalized gap	1%	1.1%	0.1%	10%

Source: Authors' estimate from 2004/2005 Household Survey

Looking at some of the differences between individuals classified as poor according to the national poverty line (Table 12), we find that poor households tend to spend a lower share of their resources on health (4.7% vs. 7.3% for the non-poor), but interestingly, they are also less likely to suffer catastrophic health payments (2% vs. 6.4% for non-poor). The out-of-pocket expenditures on health are also unsurprisingly lower for individuals below the poverty line: on average, an individual in poverty spends close to LL78,000 on health a year whereas the non-poor average is around LL403,000 per year. This is further confirmation of the result we found in Table 7 that health is a normal good.

**Table 12:** Health spending profile of the poor and non-poor

	Poor ($4.4 per day)	Non-poor ($4.4 per day)	Total
Share of spending on healthcare	4.7%	7.3%	6.6%
Catastrophic health payments	2.0%	6.4%	5.2%
Out of pocket expenditures per head (LL)	78,211	403,078	313,796

Source: Authors' estimate from 2004/2005 Household Survey

#### b. Poverty analysis using a "non-health" poverty line

We define a hypothetical poverty line for non-health expenditures using the share of health in expenditures of the average household (6.6%), and find a poverty line of $2.9 per person per day on non-health expenditures. In Table 13, we obtain a headcount of 15.4%, almost identical to the poverty rate of 15.6% when the midpoint of the upper and lower poverty lines is used. The poverty is deeper when health expenditures are included but the difference is negligible (LL6,000 per year).

**Table 13:** Non-health poverty line

	$3.3 per day poverty line	$2.9 per day non-health poverty line
Poverty headcount	15.6%	15.4%
Poverty gap (LL)	61,401	55,376
Normalized gap	3.6%	3.5%

Source: Authors' estimate from 2004/2005 Household Survey

The more interesting analysis to be conducted using this new poverty line is to try to identify individuals who fall under one of these lines but above the other. We call *health-induced *poverty the poverty of individuals whose non-health expenditures put them above the non-health expenditures poverty line, but whose overall expenditures fall below the poverty line for total household expenditures per capita. This is in line with the impoverishing effect of health care spending. We call *health-obscured *poverty the poverty of individuals whose non-health spending profile makes them poor, but whose health spending is substantial enough to pull them above the poverty line. Unsurprisingly, these two groups of individuals, shown in the off-diagonal cells in Table 14, have very different shares of health in their overall spending: health constitutes around 1.3% of the spending of the health-induced poor, and over 28% of the spending of the health-obscured poor. Health-induced poverty shows no overlap with the incidence of catastrophic payments, whereas health-obscured poverty is associated with very high rates of catastrophic payments (46%).

**Table 14:** Health-induced and health-obscured poverty

Non-health poverty			
*Total poverty*	Poor	Non-poor	Total
*Poor:*	Poor	***Health-induced***	
Share of spending on health	4.5%	1.3%	4.2%
Out of pocket expenditures on health (LL)	59,433	21,924	55,853
Risk of catastrophic health payments	2.2%	0	2.0%

*Non-poor:*	***Health-obscured***	Non-poor	
Share of spending on health	28.4%	6.7%	7.1%
Out of pocket expenditures on health (LL)	661,955	356,856	361,483
Risk of catastrophic health payments	46%	5.1%	5.8%

*Total:*			
Healthshare	6.5%	6.6%	
Out of pocket expenditures (LL)	109,544	350,958	
Risk of catastrophic health payments	5.8%	5.1%	

Source: Authors' estimate from 2004/2005 Household Survey

The spending profile on non-health related goods by the health-induced poor is comparable to that of individuals above the poverty line. It is the shortfall in their spending on health that pushes them into poverty.

In any analysis of the welfare effects of health payments, the health-obscured poor is a group that deserves attention if we are concerned about the validity of our methodology for measuring poverty: if we abstracted from healthcare payments, this group's spending profile on non-health goods would put them in poverty. So not only are the health-obscured trailing behind on non-health consumption, they are also spending more on healthcare than the typical "poor" household, which at once, obscures their poverty status and indicates that they are incurring large health expenses which is cause for concern in its own right. Thus, conventional poverty analysis fails to capture households whose poverty is health-obscured. To the extent that these households' spending on health hinders their ability to spend on other goods, health spending has an impoverishing effect on these households. While an-income based approach to poverty analysis would be able to detect the poverty of health impoverished households, this effect is missed by any expenditure based poverty analysis, as households whose health spending is substantial will show up as non-poor even when their spending on non-health goods is low.

### The equity implications of health financing methods

In this section we compare the insurance type by wealth (using wealth quintiles and poverty headcounts), in an attempt to answer the question: who is insured and what type of plan do they have?

The results obtained, are analyzed by contrasting the quality of services provided by either class of plan. As a last task in this section we investigate the effect of the type of plan on the amount spent on health.

#### a. Who is insured and what type of plan do they have?

**Figure 7:**
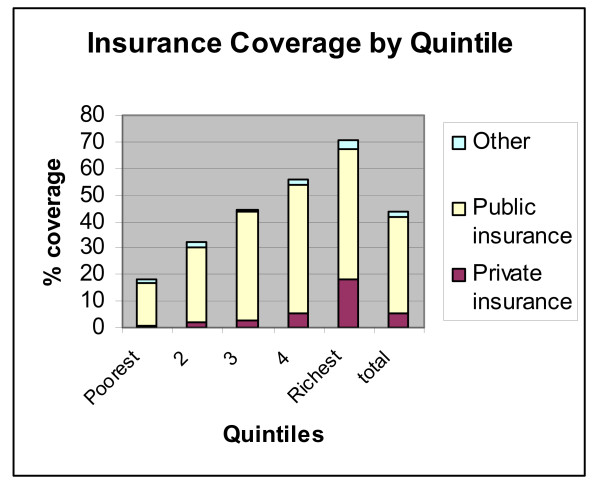
**Insurance coverage by expenditure quintile**. Source: Authors' estimates using 2004/2005 Household Survey

We first examine the difference in the coverage rates across expenditure quintiles (Figure 7): only 18% of the poorest fifth are insured and the proportion rises monotonically across quintiles and reaches just over 70% for the richest 20% of the population.

Among the insured, Figure 7 shows that the breakdown across insurance schemes also changes across expenditure quintiles: publicly provided insurance is the dominant form of insurance for all but the top quintile and it represents an overwhelmingly large fraction of the insurance plans of the third quintile (close to 92%). Similarly, the rate of private coverage is around 5% of the insured in the bottom 60^th ^percentile, rises to 10% for the next quintile and accounts for a quarter of the insured in the top fifth of the expenditure distribution.

**Figure 8:**
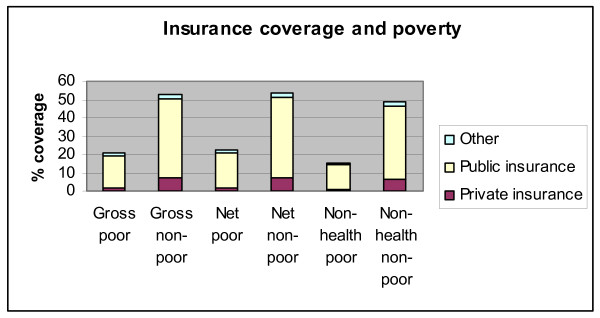
**Type of insurance and poverty**. Source: Authors' estimates using 2004/2005 Household Survey

Figure 8 looks at the crossover of the poverty headcount with the insurance coverage variable, for both the gross and net poverty headcounts as well as the "non-health" poverty that we defined earlier: consistent with the results in Figure 7, Figure 8 shows that poverty significantly reduces insurance coverage for both private and public insurance. The difference between poor and non-poor is similar for both the gross and the net headcounts. The "non-health" poverty line defines sharper divides: the "non-health" poor have lower levels of all types of insurance than the poor under either of the other poverty lines. An interpretation of this result is that once we identify as poor people who appear above the conventional expenditure poverty line because of large expenditures on health, and we identify as non-poor people who fall under the conventional poverty line because they fall short of making "average" health expenditures, but spend more on non-health than we would expect them to at the poverty line, the residual pool of poor households seems to enjoy less insurance coverage.

We examine the differences in use by different groups (Table 15). Observed use rates are highest for people covered by publicly provided insurance plans. However, the observed use of the uninsured is almost as high. Once use is indirectly standardized, it is individuals with private insurance coverage that are likeliest to spend on health and the uninsured that are least likely. Similarly, out of pocket expenditures on health increase for all categories once the figure is standardized on need and health determinants, but the increase is proportionately highest for individuals with private insurance.

**Table 15:** Health care use by insurance plan

	Insured			Uninsured
	Private	Public	Total	
Use	47.8%	52.9%	51.9%	52.7%
Use (ind. stand.)	55.6%	51.9%	52.5%	51.3%
OOP expenditures on health	484,125	327,687	352,051	279,822
OOP expenditures on health (ind. stand.)	498,570	329,331	354,161	286,711

Source: Authors' estimate from 2004/2005 Household Survey

**Figure 9:**
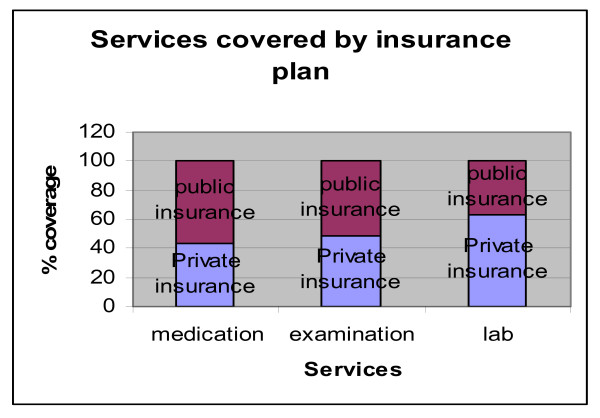
**Services covered by type of insurance plan**. Source: Authors' estimates using 2004/2005 Household Survey

The types of services that these plans cover differ substantially between privately and publicly provided insurance. Public plans offer the full range of services (medication, medical examination and lab analysis) whereas private plans only offer partial coverage of these services (Figure 9).

#### b. Hospitalization class and the quality of services

**Figure 10:**
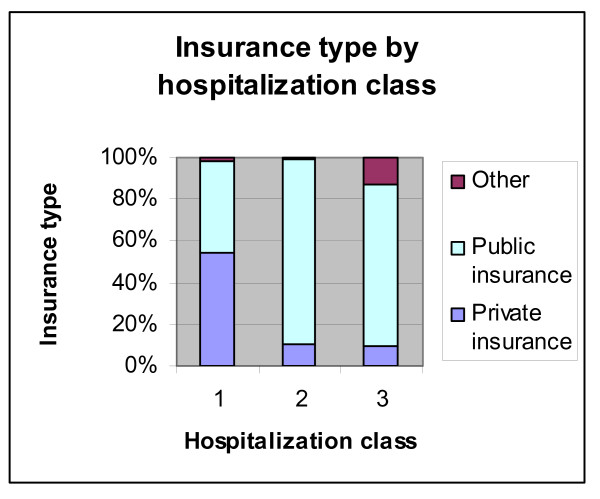
**Insurance type by hospitalization class**. Source: Authors' estimates using 2004/2005 Household Survey

These results, however, should be seen in contrast with the quality of services provided by either class of plan. Public insurance accounts for the overwhelming majority of second and third class hospitalization service, whereas private insurance accounts for more first class hospitalization plans, by a relatively thin margin of 10% (Figure 10). The quality of care is obviously strongly related to hospitalization class however we have no real measure of the magnitude of the difference in the quality of service across classes.

**Figure 11:**
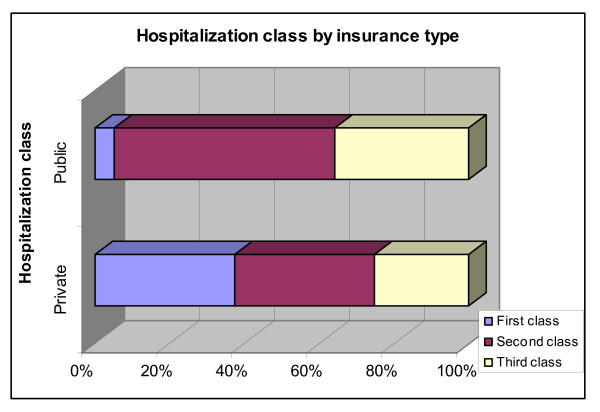
**Hospitalization class by insurance type**. Source: Authors' estimates using 2004/2005 Household Survey

The flip side of this analysis breaks down each insurance plan by class. We find that individuals with private insurance plans are concentrated in first and second class care (37% each) and only a quarter of them are eligible for third class hospitalization. Whereas only 5% of public insurance schemes provide first class hospitalization care, close to 60% provide second class care and the remaining 35% received third class care at hospitals (Figure 11).

**Figure 12:**
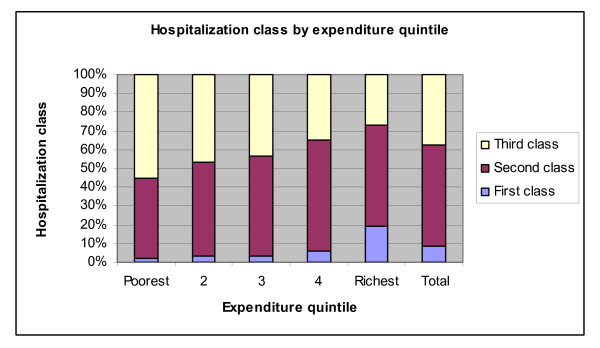
**Hospitalization class by expenditure quintile**. Source: Authors' estimates using 2004/2005 Household Survey

Finally, we look at the differences in hospitalization class across quintiles: We break down the class coverage of each quintile to find that first class hospital care is only available to 2% of the lowest quintile and to 19% of the richest quintile. Second class care is also less available to the poorest (43%) than the richest (54%), but it is the next to richest quintile that benefits the most from second class care (60%). Access to third class care progressive, and the proportion of households with third class coverage decreases secularly as we move along the distribution of expenditures (Figure 12).

**Figure 13:**
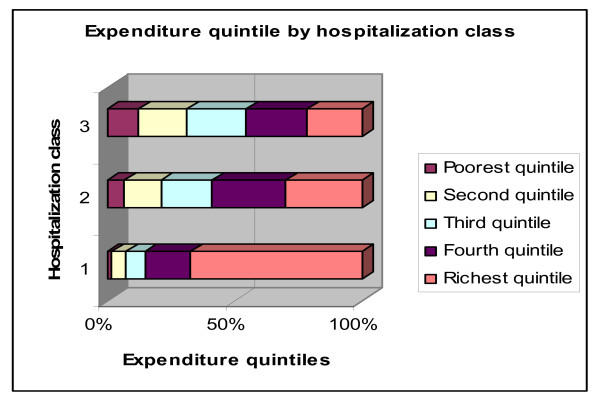
**Expenditure quintile by hospitalization class**. Source: Authors' estimates using 2004/2005 Household Survey

Figure 13 looks instead at the breakdown of each class of coverage over quintiles: the share of individuals coming from each of the first three expenditure quintiles increases monotonically as we move down hospitalization classes. Conversely, 67% of individuals with first class coverage are of the top expenditure quintile, while only 22% of individuals getting third class coverage are from the same quintile of expenditures.

**Figure 14:**
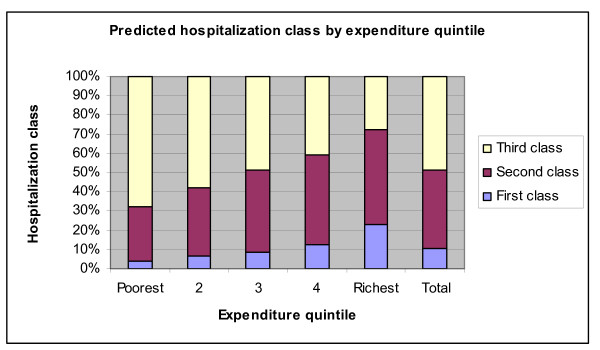
**Predicted hospitalization class by expenditure quintile**. Source: Authors' estimates using 2004/2005 Household Survey

Next we estimate a multinomial logit model of the class of hospitalization based on demographics (age, gender), insurance coverage (the presence of a plan, an indicator for publicly provided insurance), the presence of a chronic health condition, and the log of household expenditures. We reported the predicted proportion of each class of coverage based on these explanatory variables and find that the probability of coverage increases monotonically from the poorest to the richest quintile for both first and second class coverage, whereas the same probability decreases secularly from poorest to richest for third class coverage. For the bottom 60% of the expenditure distribution, the predicted proportion covered increases consistently from first to third class. Whereas the top 40% are likeliest to have access to second class hospitals care (Figure 14).

#### c. Health outcomes and health behavior by insurance plan

We investigate the effect of the type of plan on the amount spent on health (Table 16). The insured seem to spend an average of almost LL93,000 on health a year in excess of the uninsured. When we break this down by type of insurance plan, we find that this discrepancy is driven by individuals with private insurance who consistently tend to spend more on health, as well as individuals covered by a couple of public funds (municipal government plans and government cooperatives). Conducting the same type of analysis for the share of spending on health, we find, conversely, that while the insured spend larger amounts on health insurance than the uninsured, they devote a slightly smaller *proportion *of their expenditures to health. Disaggregating the sample into the sources of insurance plans, we find that results are driven mostly by individuals covered by one of three plans: the army and security forces plan, self-provided private insurance and syndicate-provided private insurance. Individuals covered by any of these three plans spend a consistently smaller share of their expenditures on health on average.

**Table 16:** Expenditures on health by insurance type

	Health expenditures (LL)	Share of expenditures on health
*Public Insurance*		
Nssf	344,419	6.2%
Coop	437,239	6.3%
Armed Forces	255,486	5.2%
Municipal	581,508	12.2%

Average public	334,676	6.0%
		
*Private Insurance*		
Employer-provided	682,588	6.7%
Self employed	726,792	4%
Mutual fund	583,874	10.3%
Syndicate	461,171	4.4%

Average private	623,218	5.2%
		
Average insured	377,900	6.0%
		
Average uninsured	280,562	7.0%

Source: Authors' estimate from 2004/2005 Household Survey

**Figure 15:**
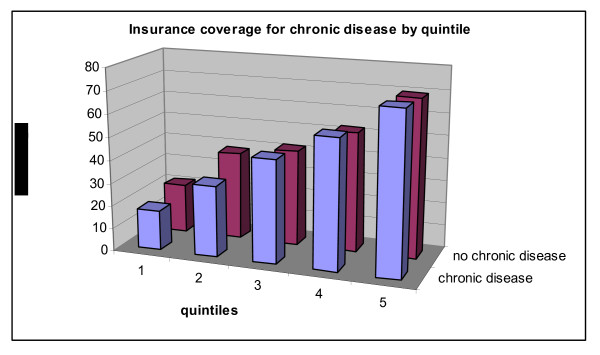
**Insurance coverage for chronic disease, by expenditure quintile**. Source: Authors' estimates using 2004/2005 Household Survey

Next, we look at insurance coverage for people with chronic health conditions, across expenditure quintiles (Figure 15). We notice an interesting reversal: for the bottom two quintiles, individuals with a chronic health condition are likelier to be insured than individuals with no chronic diseases, whereas the reverse is true for people from the top 60% of the distribution of expenditures (although the difference is negligible for the top quintile). This is likely the effect of the composition of insurance types for each quintile: the richer quintiles are likelier to have private insurance, with premiums sensitive to the underlying health condition of the insured.

### The geographical distribution of healthcare expenditures

In this section, we map our quintile analysis to geographical regions (mohafazas). We break down each mohafaza by expenditure quintile. We also investigate the difference in the incidence of disabilities and the presence of chronic health conditions across mohafazas. We then examine geographical disparities in use variables (disability cards, health expenditure, insurance coverage, hospitalization class).

#### Expenditure quintiles

**Figure 16:**
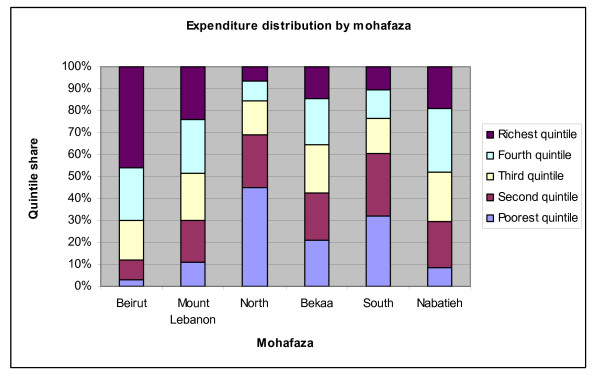
**Cumulative share of expenditures by quintiles, by Mohafaza (province)**. Source: Authors' estimates using 2004/2005 Household Survey

Beirut has the lowest concentration of the bottom two quintiles and by far the highest of the top quintile of expenditures. Mount Lebanon is also relatively well off with around half the population of the mohafaza in the top two fifths of the expenditure distribution. The poorest districts are by far the North and the South. 45% of the inhabitants of the North are in the bottom quintile of the distribution, a quarter in the next quintile and only 6.5% in the top quintile. Similarly, around a third of the residents of the South rank among the poorest fifth of the country and 60% are in the bottom two quintiles. The Bekaa is the province with the least inequality in the distribution of expenditures (Figure 16).

#### Need Variables

Disabilities are more prevalent in the Bekaa and the south of the country and least in the North. Chronic conditions, on the other hand, are significantly more prevalent in Beirut than elsewhere in the country and incidence is also lowest in the North. This may be a result of more chronic diseases getting diagnosed in Beirut than elsewhere in the country, but the data do not allow us to verify this proposition (Table 17).

**Table 17:** Disabilities and chronic disease by Mohafaza (province)

*Mohafaza*	Incidence of disability	Presence of chronic disease
Beirut	1.7%	25.8%
Mount Lebanon	1.9%	16.3%
North	1.2%	12.8%
Bekaa	2.5%	13.4%
South	2.9	18.8%
Nabatieh	2.8%	17.0%

Total	2.0%	16.2%

Source: Authors' estimate from 2004/2005 Household Survey

#### Use Variables

We restrict our attention to people with disabilities and look at the geographical distribution of people with disability cards from the Ministry of Social Affairs as well as the geographical distribution of insurance coverage. Beirut and Nabatieh have the highest subscription rates for the disabilities card (at around 45%) whereas in the Bekaa, close to only a quarter of the disabled hold disabilities cards from the Ministry of Social Affairs. Insurance coverage for the disabled is highest in Beirut, but doesn't exceed 43%, and it is lowest in the Bekaa at less than 25% (Table 18).

**Table 18:** Disability card and insurance coverage for the disabled, by Mohafaza (province)

*Mohafaza*	Disability card	Insurance
Beirut	44.3%	42.8%
Mount Lebanon	37.8%	35.1%
North	41.0%	29.7%
Bekaa	27.6%	24.8%
South	34.0%	28.1%
Nabatieh	46.4%%	20.7%

Total	36.8%	29.9%

Source: Authors' estimate from 2004/2005 Household Survey

Use variables also show large geographical variation (Table 19): Nabatieh has the highest incidence of healthcare expenditures and the highest value of healthcare spending of any mohafaza, but once standardized (indirectly), Beirut becomes a very close second. The north has the lowest rates of use and amounts spent, both observed and standardized.

**Table 19:** Incidence of use and OOP expenditures by Mohafaza (province) Source: Authors' estimate from 2004/2005 Household Survey

*Mohafaza*	Use	**Use ind. stand**.	OOP on health (in LL)	**OOP on health ind. stand**. (in LL)
Beirut	53.1%	57.8%	580,691	550,581
Mount Lebanon	49.0%	49.1%	300,007	303,848
North	45.8%	48.4%	133,166	162,776
Bekaa	56.2%	54.1%	193,053	199,417
South	58.5%	52.8%	348,312	348,343
Nabatieh	63.5%%	58.2%	649,036	651,567

Total	52.4%	51.9%	313,796	319,344

Source: Authors' estimate from 2004/2005 Household Survey

**Figure 17:**
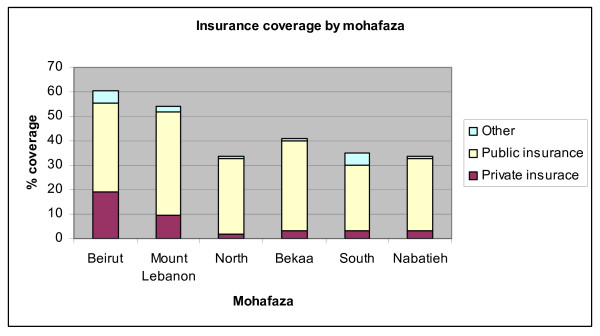
**Insurance coverage by Mohafaza (province)**. Source: Authors' estimates using 2004/2005 Household Survey

Insurance coverage shows large geographical disparities (Figure 17), with Beirut and Mount Lebanon showing 60% and 54% coverage respectively, but other mohafazas (namely the North, South and Nabatieh) trailing around 34%. The prevalence of private insurance is highest in Beirut and Mount Lebanon. Beirut has by far the highest incidence of private insurance (close to a third) and by far the lowest of public insurance (just over 60%). Mount Lebanon has the next highest rate of private insurance at around 17% and about three quarters of the insured in Mount Lebanon have publicly provided insurance. The North, the Bekaa and Nabatieh show similar patterns of rare private insurance (under 10%) and a large predominance of public insurance (close to or over 90%).

**Figure 18:**
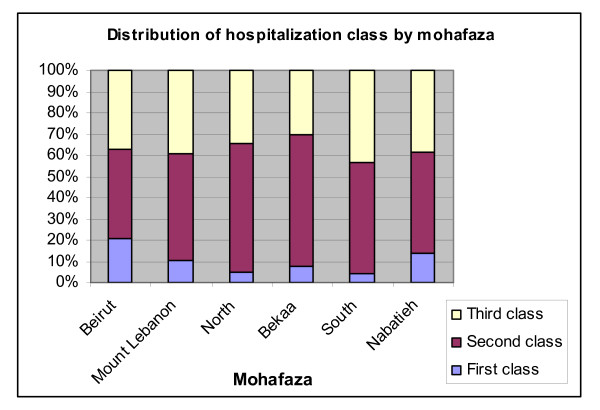
**Distribution of hospitalization class by Mohafaza (province)**. Source: Authors' estimates using 2004/2005 Household Survey

Hospitalization class also shows large disparities geographically (Figure 18): a fifth of the insured in Beirut have access to first class coverage and another two fifths have second class care. Only 4% of the insured in the South and 5% of those in the North have access to first class care. Second class care constitutes the bulk of coverage for all mohafazas, over 60% in the North and the Bekaa.

## Conclusions

The results of our analysis highlight the vulnerability of the lowest quintile of expenditures per adult equivalent, particularly when measures of healthcare use are standardized for need and demographic and economic health determinants. Not only is the healthcare use of the lowest quintiles through spending on healthcare substantially lower, they also appear to have less access to non-spending healthcare services as they are far less likely to benefit from health insurance.

Our analysis of the effect of insurance on health spending shows that once use, out of pocket expenditures and the share of spending on health are standardized, the uninsured spend both less money on health and a larger proportion of their total expenditures on health, further confirming our results on the extreme vulnerability of the lowest quintiles.

Furthermore, we take note of the weakness of running expenditure-based poverty analysis when healthcare use is also primarily measured through expenditures on health and we adjust our poverty calculations by introducing a measure of poverty that abstracts from health payments. This new measure allows us to hone in on segments of the expenditure distribution that are incorrectly labeled as poor or non-poor under the standard total expenditure based approach. Any welfare and equity analysis of healthcare reforms should correct such misclassification.

Our results call for a serious reconsideration of the targeting of health financing in Lebanon, as the lack of formal health insurance for the poorest strata of the Lebanese society makes it disproportionally exposed to adverse conditions, especially in times of conflict and instability. As it stands, the uninsured in Lebanon can benefit from medical care and hospitalization at the expenses of the Ministry of Public Health, either by going to public hospitals or by seeking preadmission to private ones, where services' payments are subject to a predetermined ceiling. But the Ministry's mandate in terms of coverage is ad-hoc and there are issues with the control of patient flows across various levels of the health care system [10]. The Ministry has limited ability to direct any of the uninsured to its own hospitals and has no way of knowing (in advance) of its full financial liability for providing these inpatient benefits. Because the Ministry cannot turn away any uninsured patients (except for those going to private hospitals who may not be guaranteed admission if annual budget ceilings have been reached), its total current (and future) expenditures on hospital care are unpredictable. Payment ceilings at private hospitals, indeed, sometimes can be exceeded when hospitals successfully petition the Ministry of Finance for payment after services have been provided.

The Ministry of Public Health in Lebanon is now responsible for the hospital care of more than half the population. It is implicitly liable for the more expensive care required by the rising number of retired and elderly persons who are not covered under any other insurance fund. As our results have shown, there is a need to revise the Ministry's strategy of covering health care for the uninsured by designing an efficient and inclusive health insurance system, which could have at least two pillars: one that covers critical and essential healthcare for the Lebanese at no cost, and an additional fully-funded pillar where insurance contributions would be proportional to income. This health insurance scheme would not only insure equity, but will also reduce in the long run the out-of-pocket expenditures of the Lebanese households.

## Competing interests

The authors declare that they have no competing interests.

## Authors' contributions

All authors have read and approved the final manuscript.

NS conducted the analysis of equity in health care utilization, the impact of catastrophic payments and the poverty implications of health care expenditures, including devising a non-health related poverty index based on expenditures data and measures of "health obscured" poverty.

JC looked at the equity implications of existing health financing methods and of existing payments for different groups of the population and conducted the quintile based analysis of various health characteristics by geographic region.

FR developed the structure of the project, secured access to the data, and supervised the various stages of the research, giving advice to the other two authors at critical junctures of the project.

## Supplementary Material

Additional file 1 **Data appendix**. A listing of chronic conditions and disabilities. Click here for file
