# Supplementary material for: Health equity in Lebanon: a microeconomic analysis
Source: Int J Equity Health. 2010 Apr 14;9:11. doi: 10.1186/1475-9276-9-11 (PMC2864280; doi:10.1186/1475-9276-9-11)
Supplement: Additional file 1 — Data appendix. A listing of chronic conditions and disabilities. [file 1475-9276-9-11-S1.DOC]

**Data Appendix**

**Chronic conditions:**

Asthma, rheumatism and rheumatoid arthritis, hypertension, migraines or chronic headaches, diabetes, epilepsy, heart disease, cancer, gastric or intestinal ulcer, diseases associated with old age,[[1]](#footnote-2) glaucoma, endocrine problems, kidney failure, liver dysfunction, cholesterol and triglycerides, prostate, chronic skin conditions, AIDS, multiple sclerosis, nervous or psychological conditions, thalassemia, osteoporosis, obesity, occupational conditions.[[2]](#footnote-3)

**Disabilities:**

Any physical or mental condition that has lasted at least 6 months and limits the respondent’s ability to perform daily activities that are normal for the respondent’s age. Disabilities are classified as: motor, speech/hearing, visual or mental.

1. These include Alzheimer’s disease, Parkinson’s disease… [↑](#footnote-ref-2)
2. These include calcification, back and neck pain, disc… [↑](#footnote-ref-3)
